# Complete genome sequence of a virulent barcoded *Mycobacterium tuberculosis* str. Erdman commonly used for non-human primate infection studies

**DOI:** 10.1128/mra.01232-24

**Published:** 2025-02-18

**Authors:** Maximilian G. Marin, Michael R. Chase, Natalia Quinones, Shoko Wakabayashi, Douaa Mugahid, Sarah M. Fortune, Maha R. Farhat, Michael C. Chao

**Affiliations:** 1 Department of Biomedical Informatics, Harvard Medical School, Boston, USA; 2 Department of Immunology and Infectious Diseases, Harvard T H Chan School of Public Health, Boston, Massachusetts, USA; 3 Broad Institute of MIT and Harvard, Cambridge, Massachusetts, USA; 4 Ragon Institute of MGH MIT, and Harvard, Cambridge, Massachusetts, USA; 5 Pulmonary and Critical Care Medicine, Massachusetts General Hospital, Boston, USA; Portland State University, Portland, Oregon, USA

**Keywords:** *Mycobacterium tuberculosis*, genome analysis, virulence, Oxford nanopore sequencing, Illumina sequencing

## Abstract

*Mycobacterium tuberculosis* is the etiologic agent of tuberculosis (TB), which claims over a million lives per year. Here, we report the complete genome sequence of a virulent barcoded *M. tuberculosis* Erdman strain that has been used extensively for non-human primate infection research studies, characterized using long- and short-read technologies.

## ANNOUNCEMENT

*Mycobacterium tuberculosis* (*Mtb*) is the bacterial agent of tuberculosis, the top communicable cause of death worldwide (1). The non-human primate (NHP) model of tuberculosis—which utilizes low-dose aerosol infection with a *M. tuberculosis* Erdman strain that was passaged through mice to maintain virulence—closely resembles the course of human infection (2) and is increasingly used to uncover mechanisms of protection against *Mtb* (3). We previously generated an isogenic library of *Mtb* Erdman containing chromosomally integrated barcodes (4), which has been subsequently used to study *in vivo Mtb* infection dynamics across a multitude of NHP studies (5–7). As *in vitro* passaging can lead to mutations (8), we provide a complete genome sequence (Erdman_SF2024) of this virulent barcoded Erdman strain that has been used for the aerosol infection of NHPs in the aforementioned studies.

An aliquot of the frozen master library (NR-50781, BEI resources) used for NHP infections was plated on 7H10 agar at 37°C for 3 weeks, and a single colony was then grown in 7H9 broth with Middlebrook Oleic Albumin Dextrose Catalase (OADC) supplementation for a week, followed by bacterial genomic DNA purification using phenol–chloroform extraction (see extended methods on Github). DNA was commercially sequenced using long-read (Oxford Nanopore) and short-read (Illumina) platforms (SeqCenter, Pittsburgh). Illumina sequencing libraries were prepared using the Illumina DNA Prep kit following the standard protocol and sequenced on a NextSeq 2000, generating a total of 29,432,744 reads (2 × 151bp paired-end). Adapter trimming was performed using bcl-convert (v4.0.3). Nanopore sequencing libraries were prepared using the ONT Native Barcoding Kit 24V14 (SQK-NBD114.24) and sequenced on a MinION device with R10.4.1 flow cells. Basecalling with Guppy (v6.4.6) and trimming with porechop (0.2.3_seqan2.1.1) yielded 104,882 reads with median length of 1,351bp and an N50 of 8,928.

The nanopore reads were *de novo* assembled into a closed, circular chromosome using the Flye assembler (v2.9) and medaka polisher (v1.9.1). The resulting long-read assembly was further polished with short reads using the PolyPolish software (v0.5) and rotated to start at the dnaA locus using Circlator (v1.5). The final genome assembly, 4,416,075 bp with 65.61% GC content, was annotated by lifting over high-confidence annotations from the H37Rv genome (GenBank AL123456.3) using RATT (v1.5), with unannotated regions filled using *de novo* annotations from Bakta (v1.9). The resulting annotated genome contains 4,011 coding sequences and harbors a barcoding plasmid at the L5 phage integration site at genomic positions: 2,764,911–2,770,126.

Erdman_SF2024 was phylogenetically mapped to the *Mtb* Erdman sub-lineage L4.1.2.1 by TB-Profiler (v6.3.0) and had fewer predicted indels and pseudogenes (Table 1)—including within putative essential genes (9) compared with another *M. tuberculosis* Erdman reference genome, ATCC35801 (10). The sequence at these discordant sites was identical between Erdman_SF2024 and H37Rv, suggesting these might represent assembly errors in ATCC35801. Finally, we performed a “mappability” analysis based on sequence similarity to define repetitive regions that could reflect areas with lower alignment confidence in Erdman_SF2024 (see Github page); these regions can be masked to increase the accuracy of short-read alignment-based variant calling in future work (11).

**TABLE 1:** Comparison of essential genes between Erdman_SF2024 and Erdman_ATCC35801

Genome	Internal stop	Frameshift	Intact	Total	Percent error
Erdman_ATCC35801	3	1	484	488	0.8196721311
Erdman_SF2024	0	0	488	488	0
Affected genes	Rv3006, Rv0337c, Rv1014c	Rv0292			

## Data Availability

All relevant code and methods are available on GitHub: https://github.com/maxgmarin/erdman-asm-explore. Sequencing reads are on SRA under accession numbers SRX26089191 and SRX26089190, both of which are associated with BioProject PRJNA1161419. The complete genome sequence and annotation are available on GenBank under accession CP172229.
